# Solitary cutaneous mastocytoma in an infant with Down syndrome

**DOI:** 10.1016/j.jdcr.2026.06.032

**Published:** 2026-06-20

**Authors:** Fatmah Altaweel, Alsadat Mosbeh, Hayam Alenezi, Abeer Albazali

**Affiliations:** a Dermatology Department, Farwaniya Hospital, Ministry of Health, Kuwait City, Kuwait; b Department of Dermatology/Dermatopathology, Faculty of Medicine, Al-Azhar University, Cairo, Egypt; c Dermatology Department, Adan Hospital, Ministry of Health, Kuwait City, Kuwait

**Keywords:** Down syndrome, mastocytoma, mastocytosis, pediatric

## Introduction

Mastocytoma represents a localized form of cutaneous mastocytosis, characterized by the accumulation of mast cells in a single area of the skin. It typically manifests in infancy as one or a few firm, brownish to yellowish papules, plaques or nodules. When the lesion is rubbed, it often develops localized redness and swelling, a reaction known as Darier’s sign is seen. The condition is benign, with most cases resolving spontaneously.^1^ Mutations in *KIT* proto-oncogene receptor tyrosine kinase (KIT) have been seen in some cases.^2^

Down syndrome, or trisomy 21, is the most common chromosomal disorder.^3^ However, mast cell disorders such as Mastocytoma are exceptionally rare in this population.

We present a rare case of solitary Mastocytoma in a one-year-old boy with Down syndrome.

## Case presentation

The patient, a 1-year 9-months-old boy, recently diagnosed with down’s syndrome and bronchial asthma, developed a yellowish to brownish plaque on his left arm.

The patient presented with a 6 months history of localized plaque on the extensor aspect of the left arm. The lesion gradually increased in size but remained asymptomatic, with no associated itching, pain, or preceding trauma. There was no history of similar lesions elsewhere or known triggering factors such as friction, rubbing, or heat exposure.

There were no associated constitutional symptoms such as fever, malaise, or weight loss. The patient had no history of recurrent infections, prolonged cough, gastrointestinal disturbances, or changes in appetite. No ocular, oral, or genital lesions were noted.

The antenatal period was uneventful, with no reported maternal illnesses or complications. The patient was born at term, normal vaginal delivery, with a birth weight of 3.2 kg. There were no perinatal complications.

Chromosomal analysis was performed shortly after birth and confirmed the diagnosis of Down syndrome, demonstrating a karyotype of 47,XY,+21, consistent with complete (non-mosaic) trisomy 21.

The patient is the third child of healthy parents, with 2 older siblings reported to be well. The parents are distant cousins. The mother was 30 years old during the pregnancy. On the maternal side, 1 cousin has a child with down syndrome, and another cousin from the paternal side of the mother’s family also has a child with down syndrome.

On examination, the patient appeared well, active, and in no apparent distress. Growth parameters were within the expected range for his age and Down syndrome. No pallor, jaundice, or lymphadenopathy was noted. There were no hepatosplenomegaly or other systemic abnormalities on general physical examination.

**Fig 1:**
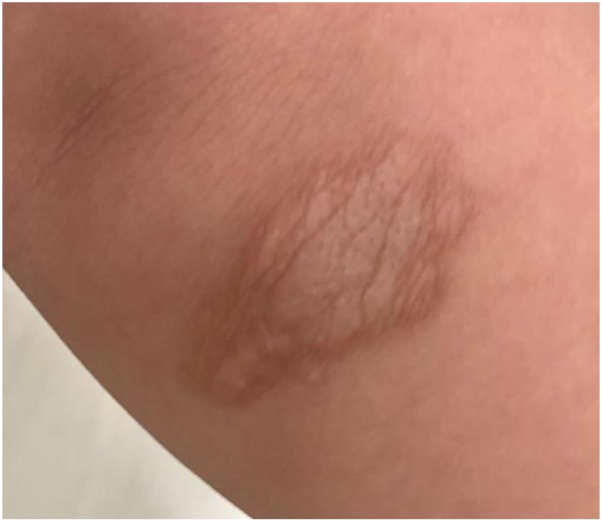
Brownish plaque measuring approximately 2 to 3 cm in diameter is noted on the extensor aspect of the left arm.

A single well-defined, oval-shaped firm, brownish plaque measuring approximately 2-3 cm in diameter is noted on the extensor aspect of the left arm. The surface appears slightly thickened with a rough texture and mildly wrinkled with an irregular border. No overlying scale, crusting, or ulceration was observed. Surrounding skin appears normal [Fig 1].

Dermoscopy revealed hypopigmented center with brownish pigmentation at the periphery. Fine linear vessels were noted. Darier sign was negative.

Routine laboratory investigations, including complete blood count, liver and renal function tests, and lipid profile were within normal limits.

**Fig 2:**
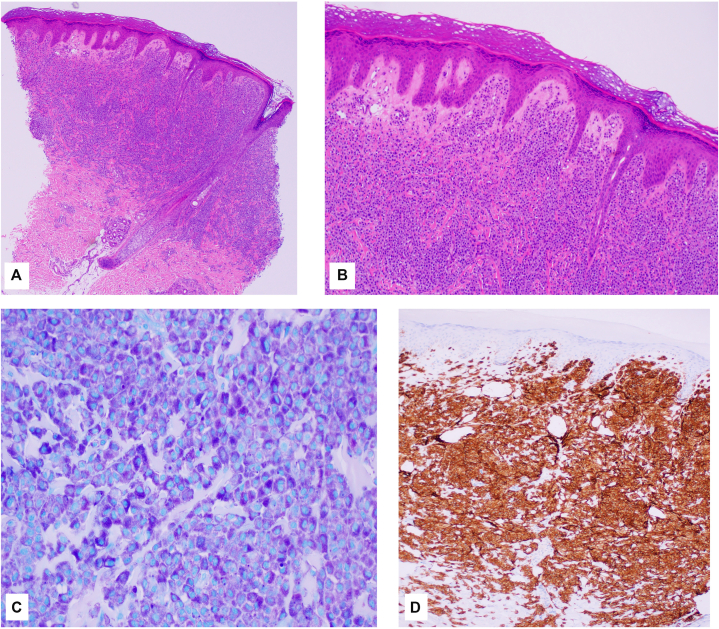
**A, B,** H&E 4× and 10×: Dense mast cell infiltrate admixed with few eosinophils in upper and mid dermis. **C,** 40×: Mast cells with metachromatic granules by Giemsa stain. **D,** 4×: Mast cells are positive for CD117.

A skin biopsy from the left elbow 0.5 × 0.5 × 0.6 cm was obtained. The specimen was bisected and processed in 1 cassette. Lesion revealed a dense, well-circumscribed dermal infiltrate composed predominantly of mast cells with round to oval nuclei and abundant granular cytoplasm. The overlying epidermis showed mild acanthosis and basal hyperpigmentation. The mast cells demonstrated metachromatic granules on toluidine blue staining and were positive for CD117 on immunohistochemistry, confirming the diagnosis of cutaneous Mastocytoma [Fig 2].

Given the patients age and underlying condition, the family preferred a conservative approach and declined surgery. Therefore, patient was managed with topical tacrolimus 0.03% and close observation.

## Discussion

Cutaneous Mastocytoma occurrence in patients with genetic syndromes such as Down syndrome is exceedingly uncommon, and no cases have been reported in the literature.

The present case shares several features with classical pediatric Mastocytoma: onset in infancy, a solitary plaque on an extremity, absence of pruritus or systemic symptoms, normal basic laboratory investigations and characteristic histology with mast cells highlighted by special stains and CD117 immunohistochemistry. Serum tryptase was not obtained as the lesion was clinically localized and there were no systemic features. Dermoscopic findings of central hypopigmentation with peripheral brownish pigment and fine linear vessels are also consistent with previously described vascular and pigment network patterns in Mastocytoma, although dermoscopy in this entity remains less well standardized than in other pigmented lesions. What distinguishes this child is the coexistence of complete trisomy 21 and the lack of any systemic features or hematological abnormalities, in contrast to earlier reports where mast cell disease in Down syndrome tended to be more extensive or associated with systemic involvement.^4^

Down syndrome is the most common chromosomal disorder and is associated with a characteristic spectrum of cutaneous manifestations, including xerosis, atopic dermatitis, alopecia areata and increased susceptibility to cutaneous infections.^3^

Although a single case cannot establish causality, the coexistence of mastocytoma and Down syndrome is noted. Trisomy 21 is associated with altered cytokine profiles, chronic low-grade inflammation, and an increased risk of certain hematologic malignancies.^5^ In pediatric mastocytosis, activating mutations in KIT have been identified in a subset of patients, but they appear less frequent and less stereotyped than in adult systemic disease, and there is currently no clear evidence that trisomy 21 directly modulates KIT-driven pathways.^1^^,^^5^

From a practical standpoint, this case reinforces that persistent, solitary brownish plaques in infants, including those with known genetic syndromes, should prompt consideration of mastocytoma and where clinical doubt exists, histological confirmation rather than attributing all cutaneous findings to the underlying chromosomal disorder should be considered.

## Conclusion

This case highlights a rare coexistence of solitary cutaneous mastocytoma and Down syndrome in early childhood. However, complete trisomy 21 is not linked to mast cell proliferative disorders, therefore, mastocytoma should be considered in the differential diagnosis of persistent pigmented plaques even in syndromic children.

Clinicians should maintain a high index of suspicion for mastocytoma in infants with solitary, slowly enlarging brownish plaques confirming the diagnosis histologically when needed, and providing appropriate counseling on trigger avoidance and follow-up.

## Conflict of interest

None disclosed.
